# Dietary Supplementation of Inorganic, Organic, and Fatty Acids in Pig: A Review

**DOI:** 10.3390/ani10101740

**Published:** 2020-09-25

**Authors:** Giulia Ferronato, Aldo Prandini

**Affiliations:** Department of Animal Sciences, Food and Nutrition (DIANA), Faculty of Agriculture, Food and Environmental Science, Università Cattolica del Sacro Cuore, Via Emilia Parmense 84, 29100 Piacenza, Italy; aldo.prandini@unicatt.it

**Keywords:** acids, feed additives, pig health

## Abstract

**Simple Summary:**

The role of acids in pig feed strategies has changed from feed acidifier and preservative to growth promoter and antibiotics substitute. Since the 2006 European banning of growth promoters in the livestock sector, several feed additives have been tested with the goal of identifying molecules with the greatest beneficial antimicrobial, growth-enhancing, or disease-preventing abilities. These properties have been identified among various acids, ranging from inexpensive inorganic acids to organic and fatty acids, and these have been widely used in pig production. Acids are mainly used during the weaning period, which is considered one of the most critical phases in pig farming, as well as during gestation, lactation, and fattening. Such supplementation generally yields improved growth performance and increased feed efficiency; these effects are the consequences of different modes of action acting on the microbiome composition, gut mucosa morphology, enzyme activity, and animal energy metabolism.

**Abstract:**

Reduction of antibiotic use has been a hot topic of research over the past decades. The European ban on growth-promoter use has increased the use of feed additivities that can enhance animal growth performance and health status, particularly during critical and stressful phases of life. Pig farming is characterized by several stressful periods, such as the weaning phase, and studies have suggested that the proper use of feed additives during stress could prevent disease and enhance performance through modulation of the gastrointestinal tract mucosa and microbiome. The types of feed additive include acids, minerals, prebiotics, probiotics, yeast, nucleotides, and phytoproducts. This review focuses on commonly used acids, classified as inorganic, organic, and fatty acids, and their beneficial and potential effects, which are widely reported in the bibliography. Acids have long been used as feed acidifiers and preservatives, and were more recently introduced into feed formulated for young pigs with the goal of stabilizing the stomach pH to offset their reduced digestive capacity. In addition, some organic acids represent intermediary products of the tricarboxylic acid cycle (TCA), and thus could be considered an energy source. Moreover, antimicrobial properties have been exploited to modulate microbiota populations and reduce pathogenic bacteria. Given these potential benefits, organic acids are no longer seen as simple acidifiers, but rather as growth promoters and potential antibiotic substitutes owing to their beneficial action on the gastrointestinal tract (GIT).

## 1. Introduction

Since 2006, the European ban of antibiotic growth promoters has been ratified by the Regulation (EC) n. 1831/2003 on additives for use in animal nutrition [1]. As reported by the Food and Agriculture Organization of the United Nations (FAO) “Animal Production and Health Division (AGA)”, the use of antimicrobial agents is essential, but the development of antimicrobial resistance (AMR) is a critical point for both animal and human health. The identification of alternative agents that can promote growth, improve feed efficiency, and reduce enteric diseases in pig farming has become a hot topic, with researchers seeking to reduce antibiotic use, while continuing to prevent pathologies and reduce mortality, so as to improve economic and environmental outcomes.

Pig farming is characterized by several critical phases; some, such as weaning or any other major change (i.e., in management, feeding strategy or environment), can cause stress and disturb the optimal physiological status. These stresses are often reflected by the appearance of gastrointestinal disorders and worsening of growth performance and feed efficiency. Gastrointestinal tract (GIT) plays a key role in achieving growing performance. Thus, modulating the health and functionality of the GIT mucosa through feeding strategies could reduce disease appearance and, consequently, decrease antibiotic use. For example, weaning piglets are characterized by under-developed digestive capacity and intestinal microbiota, and feed additives could be used to promote growth performance and prevent enteric diseases. Growing evidence suggests feed additives should also be given to fattening pigs, not only to improve performance, but also to reduce nitrogen excretion, phosphorous excretion, and the environmental impact [2].

Feed additives may be classified into the following groups: acids, minerals, prebiotics, probiotics, yeast, nucleotides, and phytoproducts [3]. Among them, acids offer many beneficial properties. Acid are molecules able to donate protons and are characterized by a dissociation constant and acidifier power that influence its effect. Acids may be administered as feed additives in various ways, including as a pure form, as a blend of organic and/or inorganic acids, or in association with phytoextracts or enzymes that can enhance the beneficial impacts. The group of feed additives categorized as acids includes inorganic acids, organic acids, fatty acids, and their salts. The members of the organic acid group may be classified by their saturation level (saturated or unsaturated) and/or carbon chain length (short, medium, or long chain) [4]. Some acids, like volatile fatty acids (VFAs) or intermediate products of tricarboxylic acid cycle, are naturally produced at the enteric level as a result of digestive processes, and/or at the cellular level [5].

Acids have long been used as feed acidifiers and preservatives; more recently, they have been included in feed stock for young pigs, with the goal of stabilizing the stomach pH to improve their digestive capacity [6]. Some organic acids could also be considered an energy source, such as those acting as intermediary products of the TCA cycle [2]. Moreover, the antimicrobial properties of some organic acids have been exploited to modulate microbiota populations and reduce pathogenic bacteria. These potential benefits mean that organic acids are no longer viewed as simple acidifiers of animal feed, but rather as growth promoters and potential antibiotic substitutes.

However, the effects of acid supplementation reported in the bibliography are not uniform both across different physiological phase and within the same physiological phase. Various authors have reported that the efficacy of acids can be dose-, stomach pH-, and microbiota population-dependent. Moreover, acid blends are more commonly used as feed additives, as blends tend to have wider-ranging action against pathogens than single acid formulations. Acids do not have a consistent mode of action in this context, but their beneficial effects could be due to the following:

- capacity of acids in undissociated forms to cross pathogen cell membranes and act on membrane enzymes or components of the cytoplasm (bactericidal/bacteriostatic effects);
- stabilization of the stomach pH (especially during the weaning period, when there is insufficient endogenous gastric HCl secretion) because of H^+^ ions produced by acid dissociation, reduction of intestinal pH creating a barrier and/or hostile condition against pathogen growth and colonization, and/or activation gastric pepsinogen;
- improvement of nutrient digestibility and endogenous VFA production;
- acting as an energetic substrate for epithelial mucosa cells of GIT, improving the villus crypt/depth ratio and relative absorptive capacity;
- acting as a precursor for amino acids (AAs) synthesis;
- increasing blood flow [7,8].

The aim of this review is to provide a state-of-the-art overview of the acid group of feed additives, which act not only as acidifiers, but also as promoters of intestinal health and performance, nutritional compounds, and antimicrobials.

## 2. Inorganic Acids

Inorganic acids or minerals acids are characterized by the presence of a non-metal element; they can be differentiated as hydrogen halides or halogen oxoacids depending on the presence of an oxygen molecule. Inorganic acids have been used as feed additives in part because they are much less expensive than organic acids. Most commonly used feed additive acids are hydrochloric acid (HCl), phosphoric acid (H_3_PO_4_), and sulfuric acid (H_2_SO_4_). However, the published studies have revealed discordant results on their positive effects and there are much less data on inorganic acids in this context, versus organic acids. Kil et al. reported that hydrochloric acid supplementation showed a greater beneficial effect on average daily gain (ADG) and average daily feed intake (ADFI) than organic acid supplementation, but there was no difference in growth performance [9]. These authors found that the serum level of Cl^-^ was not affected by this treatment, which countered two earlier reports [10,11]. In the first two weeks post-weaning, the production of HCl in the pig stomach is insufficient for optimal protein digestion; thus, supplementation of Cl during this period can improve performance, N retention, and digestibility. HCl production is further suppressed by the presence of *Lactobacilli spp.* in the stomach, by the milk feed and consequent lactic acid production that suppress HCl production. Two studies [12,13] showed that Cl supplementation greatly impacted the N digestibility of a starter diet containing spray-dried blood plasma or dried whey during the initial two weeks post-weaning. Supplementation with sulfuric acid reportedly carries a risk of feed efficiency depression, probably due to alteration of the electrolyte balance [14], while the inclusion of phosphoric acid was found to lower the pH, but not improve nutritive efficacy [15]. Walsh et al. reported that the use of an inorganic acid blend yielded a growth performance comparable to that of antibiotics or organic acid blends, but that its combination with an organic acid blend reduced growth performance [16]. The authors confirmed the observation in a subsequent study and suggested that blends of organic and inorganic acids might be more beneficial owing to broader-spectrum activity, even if the presence of excess acids somewhat decreased feed palatability and intake [17]. Table 1 presents additional details on the relevant studies related to feed supplementation with inorganic acids.

## 3. Organic Acids

The members of the organic acid group may be used alone or in a blend of organic acids. Each acid presents chemical properties that influence its beneficial effects, and the dose response is not always constant. Table 2 presents additional details related to these acids, including their doses and effects.

### 3.1. Lactic Acid

Lactic acid (C_3_H_6_O_3_) is colorless-to-yellow in the form of a viscous liquid or crystal; it is soluble in water and is a strong acid (pK_a_ = 3.86). It is naturally produced in pig stomach and small intestine as an end product of sugar fermentations and lactate. Its salt form is produced by muscle cells to support pyruvate oxidation and adenosine triphosphate (ATP) production [18]. Moreover, lactic acid is produced by bacteria like *Lactobacillus*, *Bifidobacterium*, *Streptococcus*, *Pediococcus*, and *Leuconostoc*, which convert carbohydrates into lactic acid [19]. Lactic acid acts in an antimicrobial way through the reduction of gastric pH thanks to the release of H^+^ and the stimulation of pancreatic exocrine response. The presence of undissociated acid inhibits the proliferation of Gram negative bacteria (*Escherichia, Salmonella*), but does not affect the proliferation of Gram positive bacteria like *Lactobacilli* and *Bifidobacterium* that are tolerant to pH reduction, thus resulting in an increased growth performance due to the reduction of energy loss [2]. Lactic acid supplementation of feed is mostly given to suckling and weaning pigs, which have incompletely developed GIT and immune systems and may experience alterations of the microbiota composition and gut integrity at weaning [20]. Thaela et al. reported that supplementation with lactic acid stimulated pancreatic secretion in piglets after weaning [21]. Tsiloyiannis et al. reported that lactic acid was useful in controlling post-weaning diarrhea [22]. Kemme et al. tested acid lactic supplementation of growing-finishing pigs and reported that this strategy enhanced the apparent ileal degradation (AID) of N and AA; the apparent total tract digestibility (ATTD) of ash, Ca, and Mg; and AID of phytic acid, and found that this did not involve a hypothesized delay of gastric emptying, which was expected to prolong the residency of phytase in the stomach [23,24]. Lactic acid is not often used in finishing pigs because of their high digestive enzyme capacity and, concerning sow feed supplementation, only few studies are reported even if the influence of mother microbiota on piglet’s composition is noted. Tanaka et al. reported that supplementation could control clinical and sub-clinical infections of *Salmonella typhimurium* in swine [25]. Yang et al. showed that the lactic acid concentration could also be enhanced through supplementation of lactic acid bacteria, and proposed that this probiotic view could be considered a long-term solution in swine [20].

**Table 2:** Summary of studies about the most common organic acids used in pig farming.

Compound	Animal Category	Dose	Response	References
Lactic acid	Suckling-weaning pigs	2.5%	Increased volume, protein, trypsin and chymotrypsin content in pancreatic juice	[21]
	Weaning pigs	1.6%	Increased average daily gain (ADG) and average daily feed intake (ADFI). Decreased feed conversion ratio (FCR) and post-weaning diarrhea incidence	[22]
	Finishing pigs	3.0%	Enhancement of N and amino acids (AA) apparent ileal degradation and apparent total tract digestibility (ATTD) of ash, Ca, Mg, and average daily intake (AID) of phytic acid	[23,24]
	Sows	2.8%	Reduction of ST116Rif^r^ in the gram feces and liver, tonsil, mesenteric lymph nod, and jejunum/cecum content and bacteria count	[25]
Fumaric acid	Weaning pigs	0.2%	Increased N retention and reduction of urinary-N excretion	[9]
	Weaning pigs	1.5%	Greater ADG and feed conversion efficiency	[22]
	Weaning pigs	1.0% or 2.0% or 3.0%	Increased ileal digestibility of crude protein (CP), gross energy (GE) and AAs in low buffering diet, fecal histidine digestibility, ileal digestibility of GE, CP and AAs from 3 to 4 weeks after weaning	[26]
	Weaning pigs	2.0%	No effects on nutrient digestibility or gain	[27,28,29]
	Weaning pigs	1.5%	Increased gain, microbial culture, and gain to feed ratio (G/F) (inconsistent results), stomach fumarate concentration. No interactive effect with citric acid	[30]
	Weaning pigs	1.5%	No effect on postweaning performance of pig challenged with *E. coli*	[31]
	Growing pigs	0.20%	No effect on apparent digestibility of protein and dry matter	[29]
	Finishing pigs	1.0% or 2.0%	Increased ADG and FCR	[28]
	Finishing pigs	1.0% or 2.0% protected-organic acids (OA) blend (17% fumaric acid)	Increased ADG, digestibility of dry matter (DM), N and energy digestibility, longissimus muscle area, color and firmness meat, *Lactobacillus* count. Decrease of fecal ammonia, acetic acid emission, and *E. coli* count	[32]
	Weaning pigs	OA blend (3.0% calcium formate + 1.0% fumaric acid) + Essential Oils (EO)	Positive effects on intestinal health and digestive enzymes, improved ADG, digestibility of Ca, Ps, and CP, count of *Lactobacilli* into feces, villous height in duodenum and jejunum and butyric and valerianic acid concentrations in colon	[33]
Malic	Suckling pigs		No effect on ADG and ADFI	[34]
	Suckling pigs	1.2%	Beneficial effects on poste-weaning diarrhea	[22]
	Tartaric		No effects	[35]
α-ketoglutaric acid (AKG)	Weaning pigs	5.0%	AKG could replace dietary AAs and could be directly used as an energy source by enterocytes	[36]
	Growing pigs	1.0%	Beneficial effect on adenosine monophosphate (AMP)-activated protein kinase (AMPK) and energy status in the intestinal mucosa of piglets challenged with *E. coli* lipopolysaccharide (LPS)	[37,38]
	Growing pigs	1.0%	Ameliorated LPS-induced liver injury, increased antioxidant capacity, and improved energy metabolism	[39]
	Weaning pigs	1.0%	Higher serum and intramuscular AAs concentrations, mRNA abundance of AA transporters, serum concentration of IGF-I, activation of mammalian target of rapamycin (mTOR) pathway and decreased serum urea concentration and genes related to muscle protein degradation	[40]
	Weaning pigs	1.0%	Beneficial bacteria growth promotion, increased butyrate and valerate concentrations. Decreased ammonia levels in the gut	[41]
Citric acid	Weaning pigs	1.5%	Control of post-weaning diarrhea incidence	[22]
	Weaning pigs	1.5%	Inconsistent results about growth performance in piglets challenged with *E. coli*	[30,31]
	Weaning pigs	3.0% or 6.0%	Increased G/F, metatarsal ash, small effect on phytase utilization	[42]
	Growing pigs	0.4% formic-citric acids blend + EO	Increased ADG. Reduction of fecal concentration of *Salmonella*	[43]
Chlorogenic acid	Weaning pigs	0.025% or 0.05% or 0.1%	Increase of duodenal/jejunal villus height and duodenal villus height/crypt depth ratio, activity of serum and duodenal glutathione peroxidase (GSH-Px) and duodenal catalase (CAT), *Lactobacillus* population, higher propionic and butyric acid concentration in colon, G/F with 0.1% dosage and ADG, ATTD of crude protein, fat and ash, serum albumin, IGF-I and activities of superoxidase dismutase/glutathione peroxidase/ catalase (serum), maltase (jejunum/ileum), mRNA levels of sodium glucose transport protein-1/zinc transporter-2, divalent metal transporter-1 (jejunum); upregulated occludin (OCLN) duodenum/jejunum expression. Decrease of G/F ratio and duodenal crypt depth, ileal Malondialdehyde content, *E. coli* count, diarrhea incidence	[44,45]
	Weaning pigs	0.10%	Decreased serum D-lactate and diamine oxidase, mucosa histamine and typhase contents, malondialdehyde content (duodenal/jejunal mucosa); increased expression of claudin-1 protein, glutathione peroxidase and catalase, antioxidant signaling (duodenal/jejunal mucosa). Erythroid 2-related factor/ heme oxygenase-1 (Nrf2/HO-1) pathway activation and toll-like receptor 4 / nuclear factor-kappa B (TLR4/NF-kB) pathway suppression	[46]
	Weaning pigs	0.10%	Increase of jejunal villus height, duodenal/jejunal villus width, jejunal/ileus height-crypt depth. Decrease of serum tumor necrosis factor (TNF), interlukin-6, interlukin-1betaIL-6. Increase of serum immunoglobulin (Ig) G and jejunal IgA. Decrease duodenal/jejunal number cells in G_0_G_1_ phase, decrease jejunal/ileal number cells in S phase, % of late and total apoptotic cells in jejunum and apoptosis regulation (Bcl-2)-associated X protein/ Bcl-2 ratio. Upregulation of caspase-3 in duodenum/jejunum, downregulation capspase-3 in duodenum/jejunum, caspase-9 in jejunum, Fas in jejunum/ileum	[47]
	Weaning pigs	0.10%	Increase small intestine length, diamine oxidase activity and class-II major histocompatibility proteins (MHC-II) concentration in jejunal and ileal mucosa. Increased volatile fatty acids (VFA) concentration in cecum digesta and bacterial alpha diversity (*Firmicuts* and *Bacteroidetes*)	[48]
	Growing pigs	0.05%	Increased gut microbiota diversity, serum aspartic acid/threonine/alanine/arginine and colonic 5-HT	[49]
Benzoic acid	Suckling pigs	0.1% or 0.2 or 0.3% (benzoic acid + EO) and 0.5% benzoic acid	Increased average body weight (BW), ADG, and ADFI. Decrease of *Bacteroides* and *Prevotellaceae Prevotella*. Upregulation of caprolactam/limonene/pinene degradation and C5-branched dibasic metabolism; downregulation of prostatate cancer and nucleotide-binding oligomerization-like receptor pathways	[50,51]
	Weaning pigs	0.5%	Increased ADFI, ADG, ATTD of DM, CP, ethereal extracts (EE), GE and crude ash, mRNA expression of glucagon-like peptide 2, glutathione peroxidase and superoxidase dismutase activities (jejunum). Enhanced activity of trypsin, lipase and amylase and villus height to crypt depth ratio (jejunum). Reduced digesta pH and crypt depth (jejunum)	[52]
	Weaning pigs	0.5%	Increased *Bifidobacterium* (ileum) and *Bacillus* (cecum) counts, total VFA and propionic content (cecum), villus height (duodenum/ileum), villus height/crypt depth ration (duodenum/jejunum/ileum). Decreased digesta pH and *E. coli* and *Enterococci* counts, NH_3_-N concentration (cecum), crypt depth (duodenum)	[53]
	Growing pigs	1.0% or 2.0%	Increased blood pH, ATTD of Ca, P, Na; plasma P, K and Na and concentration of P in femur ash. No effect of utilization of dietary Mg. Decreased urine pH, ATTD of Cl, retention of Na and Cl, concentration of ash in femur, Ca and Cl in femur ash	[54]
	Growing pigs	0.5% or 2.5% or 5.0%	Excessive supplementation decreased ADFI, ADG, white blood cell, and globulin; enhanced serum alanine aminotransferase and aspartate aminotransferase, liver injury, splessen damage, residues in liver and kidneys	[55]
Fulvic acids	Growing pigs	0.20% fulvic acids + 0.20% probiotics	Increased P, GE and ash digestibility, sheep red blood cells (SRBC) antibody titers, IgG, IgA, phytohemaglutination	[56]
	Finishing pigs	5.0% or 10.0%	Increased ADG, G/F, lymphocyte count, and marbling score. Decreased backfat thickness	[57]
	Finishing pigs	0.2% or 0.4% or 0.6% or 0.8%	Increased HSL activity, serum low-density protein, leptin, growth hormone, insulin, triiodothyronine. Reduced backfat thickness, lipoprotein lipase (LPL) activity	[58]
	Growing pigs	0.2% or 0.4% or 0.6% or 0.8%	Increased ADG, G/F, and muscle marbling. Reduced backfat thickness, muscle pH, and malonaldehyde	[59]
	Growing pigs	3.0% sodium humate	Decreased digestibility of CP, fat, and ammonia emissions	[60]
	Weaning pigs	0.5% or 1.0%	Increased ADG and G/F, but inconsistent results across the study. Reduced ammonia emissions from manure	[61]
	Weaning pigs	2 g or 4 g/pig/day	Increased N apparent total tract digestibility. Decreased N intake, N fecal excretion, manure ammonia emissions, manure odor	[62]

AA: amino acids; ADFI: average daily feed intake; ADG: average daily gain; ADI: average daily intake; AID: apparent ileal degradation; AKG: α-ketoglutaric acid; AMP: adenosine monophosphate; AMPK: activated protein kinase; ATTD: apparent total tract digestibility; Bcl-2: apoptosis regulation; CAT: catalase; BW: body weight; CP: crude protein; DM: dry matter; EE: ethereal extracts; EO: essential oils; FCR: feed conversion ratio; G/F: gain to feed ratio; GE: gross energy; GSH-Px: glutathione peroxidase; HO-1: heme oxygenase-1; HSL: hormone sensitive lipase; Ig: immunoglobulin; LPL: lipoprotein lipase; LPS: lipopolysaccharide; mTOR: mammalian target of rapamycin; Nrf2: Erythroid 2-related factor; NF-kB: nuclear factor-kappa B; MHC-II: class-II major histocompatibility proteins; OA: organic acid; OCLN: occludin; SRBC: sheep red blood cells; TLR4: toll-like receptor 4; TNF: tumor necrosis factor; VFA: volatile fatty acids.

### 3.2. Fumaric Acid

Fumaric acid (C_4_H_4_O_4_) is a colorless crystalline compound with a fruit-like taste and a biphasic pK_a_ (3.02 and 4.44). Its salt, fumarate, is an intermediate of the citric acid cycle and also a product of the urea cycle. Even though there is some debate as to the effectiveness of fumaric acid as a feed additive, it is often used for this purpose because of its solid form and relatively low cost [18] compared with citric acid. As an intermediate product, it could be used as a quickly available energy source, effective on small intestine mucosa with a trophic effect and increasing absorbate surface [2].

Tsiloyiannis et al. reported ADG improvements in weaned piglets [22]. Giesting and Easter showed that fumaric acid supplementation had beneficial effects on ileal gross energy digestibility, as well as the levels of crude proteins and AAs [26]. However, as seen for citric acid, the published results are not consistent in this regard. For example, Blank et al. [27], Falkowski and Aherne [28], and Thacker et al. [29] reported that fumaric acid did not significantly affect the apparent digestibility of proteins and dry matter, while Risley et al. observed no alteration of the counts of *Lactobacilli* or *E. coli* in the GIT under fumaric acid supplementation [30,31]. Some authors had included various proportions of fumaric acid or its salts in organic acid blends. For example, Upadhaya et al. reported that an organic acid blend had relatively minor effects on growth performance, nutrient digestibility, and fecal gas emissions in finishing pigs [32]. In weaned piglets, Kil et al. reported that a similar dosage/blend had, as its only significant effect, the reduction of urinary-N excretion [9]. Xu et al. used a blend of fumaric acid and essentials oil, and found that this formulation had a positive effect on intestinal health [33].

### 3.3. Malic Acid

Malic acid (C_4_H_6_O_5_) is a biphasic dicarboxylic acid (pK_a_ 3.40 and 5.10) available in liquid or white crystalline powder forms; it is odorless and may have a tart taste. Its salt form, malate, is an intermediate of the citric acid cycle, and it can also be formed from pyruvate via anaplerotic reactions. This compound has not been widely investigated in the context of feed supplementation and the results are not consistent. One study failed to find performance improvement on ADG or average ADFI in nursery pigs [34], while another reported that an organic acid blend had beneficial effects on post-weaning diarrhea syndrome in piglets [22].

### 3.4. Tartaric Acid

Tartaric acid (C_4_H_6_O_6_) is a hydroxy-carboxylic acid (pK_a_ 2.89 and 4.40) that is naturally present in many fruits (e.g., grapes); it is mainly used as a food preservative and antioxidant. Notably, it is a muscle toxin that, at a high dose, inhibits malic acid production and causes paralysis and death (“DL_50_” 7.5 g/kg for human, 5.3 g/kg for rabbits, and 4.4 g/kg for mice) [63]. This compound is rarely used as an acidifier and growth promoter, also in part because it is excreted into urine after oral administration and can be degraded by intestinal microflora [35].

### 3.5. α-Ketoglutaric Acid

α-Ketoglutaric acid (AKG; C_5_H_6_O_5_) is derived from glutaric acid; its anion form, α-ketoglutarate, is produced during the Krebs cycle through glutamate deamination [64]. AKG plays a key role in systemic, intestinal, and gut bacterial metabolism; that is, it acts as an N transporter in metabolic pathways and as an antioxidant and anti-inflammatory, connects AAs metabolism with glucose oxidation [65], contributes to adenosine triphosphate (ATP) homeostasis and nitric oxide-nitric oxide synthetase balance (NO–NOS) [65], and has anticatabolic effects [66]. These actions are reflected in improved intestinal mucosa integrity and increased absorption capacity. Kristensen et al. proposed that AKG supplemented to animal diets could replace dietary dispensable AAs by shunting ammonium back into the dispensable AA pool [36]. The same authors tested AKG supplementation through the mesenteric vein in growing pigs, but found that the stomach concentration did not significantly differ from the plasma concentration, probably because AKG could be absorbed and metabolized by the stomach/duodenum epithelia, metabolized by bacteria, and/or absorbed by enterocytes. Hou et al. and Wang et al. tested dietary supplementation of AKG and found that it had beneficial effects on AMP-activated protein kinase (AMPK) and energy status in the intestinal mucosa of piglets challenged with *E. coli* lipopolysaccharide (LPS) [37,38]. The reduction of these effects of LPS alleviated the mucosal damage, improved the absorptive function of the small intestine, and activated the signaling of mTOR (mammalian target of rapamycin), which is involved in the insulin, growth factor, and AA pathways [39]. Chen et al. observed that AKG supplementation of a low-protein diet enhanced the serum and intramuscular AA concentrations, the mRNA abundance of AA transporters, and the serum concentration of insulin-like growth factor-I (IGF-I); activated the mTOR pathway; and decreased serum urea and the expression of genes related to muscle protein degradation [40]. At the same dietary dosage and in combination with allicin, Liu et al. reported that AKG enhanced the cecal microbiota composition and was associated with higher concentrations of acetate, butyrate, and total VFAs [67]. Consistent with this, Chen et al. found improved counts of *Lactobacillus* and *Bifidobacterium* in the cecum and *Lactobacillus* and *Firmicutes* in the ileum, reduced counts of *E. coli*, and a decreased concentration of ammonia in the ileum of pigs [41]. The same authors found that dietary supplementation of AKG could affect the N balance and the N-utilization rate by reducing urinary N excretion and enhancing Ca and P utilization and metabolism in growing pigs, thereby reducing the environmental impact of their production [41].

### 3.6. Citric Acid

Citric acid (C_6_H_8_O_7_) is a colorless crystalline compound with a sour taste [2]. This triphasic carboxylic acid has a relatively low pK_a_ (3.13, 4.76, and 6.34) and a chelating capacity towards metallic cations. It is also an intermediate metabolite of the tricarboxylic acid (TCA) cycle (Krebs cycle), could contribute to fatty acid synthesis, and may inhibit glycolysis at high concentrations. These characteristics mean that microorganisms can metabolize citric acid, making it a less effective antimicrobial agent [18]. Tsiloyiannis et al. showed a positive effect on controlling post-weaning diarrhea [22]. Risley et al. reported that citric acid supplementation had no significant effect on pH, VFA, non-VFA, the microbiota composition in the GIT of weanling pigs, or the *E. coli* count in post-weaning pigs [30,31]. Supplementation of citric acid in chicks yielded improved phytate P utilization; in pigs, however, a relatively higher dosage yielded a smaller effect [42]. Finally, citric acid plus formic acid was reported to reduce the fecal *Salmonella* count [43].

### 3.7. Chlorogenic Acid

Chlorogenic acid (CGA) (C_6_H_18_O_9_) is an organic acid and an ester of caffeic and quinic acids. It is a phenolic acid that is found in many plants. CGA shows anti-inflammatory properties and, in this context, has been tested as a dietary supplement for pig farming. In weaned piglets, Zhang et al. reported that CGA had dose-dependent effects on improving gut morphology, as evidenced by a better villus height/crypt depth ratio at the duodenal level associated, improved activity of serum GSH-Px, a higher count of *Lactobacillus*, a lower count of *E. coli*, and higher concentrations of propionic and butyric acid in the colon [44]. Chen et al. confirmed that CGA supplementation generally improved gut mucosa health and growth performance [45]; the authors further suggested that these changes may be related to improvement of immune defense and suppression of excessive apoptosis among intestinal epithelial cells, and that this may occur via suppression of toll-like receptor 4/nuclear factor-kappa B (TLR4/NF-кB) signaling and activation of erythroid 2-related factor/heme oxygenase-1 (Nrf2/HO-1) signaling [46,47]. CGA dietary supplementation showed a modulatory effect on microbiota populations, increasing *Lactobacillus spp*., *Prevotella spp*., *Anaerovibrio spp*., and *Alloprevotella spp*. in the cecum [48]. In growing pigs, CGA enhanced gut microbiota diversity, the serum free-AA levels (aspartic acid, threonine, alanine, and arginine), and the colonic 5-HT level [49].

### 3.8. Benzoic Acid

Benzoic acid (C_7_H_6_O_2_) is a colorless crystalline powder and is the simplest aromatic carboxylic acid. It is a weak acid with a pK_a_ value of 4.19 and is mainly absorbed and transported through the monocarboxylic acid transporter in the small intestine. Generally, benzoic acid improves nutrient utilization mainly as a result of a stimulation of digestive enzymes production and activation, enhancing the villus/crypt ratio and absorption surface and improving of gut barrier function through non-specific barrier mechanisms, specific immunological responses, and microbiota composition. Nevertheless, the potential positive effects could be affected by several factors, such as age, dietary type and composition, and environment [68]. At the European level, it has been approved as a feed additive for pig nutrition only for weaned piglets and fattening pigs. For example, Papatsiros et al. found that including acid in the feed of weaners improved growth performance, body weight (BW), and ADG, and decreased the diarrhea score and *E. coli* count [69]. This was reported to be particularly evident when associated with *Bacillus cereus* var. *toyoi* and due to a strong bactericidal effect on coliform and lactic acid bacteria. The combination of benzoic acid with essentials oils also reportedly showed clear effects. Wang et al. and Zhai et al. found that the feed intake and growth rate were improved under this supplementation, although the gain-to-feed ratio (G/F) was unchanged; the authors proposed that this could be related to regulation of microbiota rather than a change in nutrient digestibility [50,51]. According to Diao et al., better nutrient digestibility was allowed by enhanced digestive enzyme activity and mucosa absorption surface [52]. The authors suggested also that a given dose of benzoic acid yielded better nutrient digestion and jejunal morphology when co-applied with glucagon-like-peptide-2 (GLP-2), increasing production, and acid antioxidant capacity in young pigs, and maintaining the balance of microflora [53]. In fattening pig and sows, the inclusion of benzoic acid was reported to improve the utilizations of dietary Ca, P, and K; reduce those of dietary Na and Cl; and have no effect on that of Mg [54]. Given the many mechanisms involved in acid metabolism, these results could suggest that benzoic acid could be used to reduce the environmental footprint of pig production, in the case where microminerals requirements were exceeded. Regarding the suggested dose, benzoic acid should be applied in feeds, and studies have shown that excessive intake could induce dysfunction and damage of the liver, spleen, and lung, and trigger modification of gut morphology [55,68].

### 3.9. Fulvic Acids

Fulvic acids (FAs) are a group of organic acids that are naturally present in the humus (humic substances) and have an average formula of C_135_H_182_O_95_N_5_S_2_. Owing to the presence of many reactive functional groups, FAs may have antioxidant and/or chelating activities and indirectly could improve minerals’ utilization [56]. Dietary supplementation of FAs is not widely studied in the pig sector. Kunavue et al. reported that FA supplementation of weanling pigs yielded better levels of P, gross energy, ash digestibility, and (higher) serum IgG [56]. Wang et al. found that supplementation of humic substances (HS) yielded increases in ADG, G/F (HS 10%), and lymphocyte counts [57], but a decrease in backfat thickness. Chang et al. confirmed that different doses of FA reduced pig backfat, and found that this was due to a higher activity of hormone sensitive lipase (HSL) and lower activity of lipoprotein lipase (LPL) [58]. Bai et al. reported that dietary supplementation FA could increase G/F and reduce backfat thickness and malonaldehyde (MDA) in growing–finishing pigs [59]. Supplementation with sodium humate was also found to increase the digestibility of crude protein and crude fat in growing pigs; the resultant decrease in N-containing substances, in particular, could help decrease ammonia emissions [60,61,62].

## 4. Fatty Acids

Fatty acids are a category of organic acids characterized by aliphatic chains with 4–28 carbons, where the carboxylic group is hydrophilic, while the carbon chain is hydrophobic. These could be chemically classified according their carbon chain length into short chain fatty acids (SCFAs; 1–5 carbon atoms), medium chain fatty acids (MCFAs; 6–12 carbon atoms), or long chain fatty acids (LCFAs; 13–21 carbon atoms), and according to saturation level, into satured or unsatured fatty acids. These are used as a pig feed strategy owing to their antibacterial potential (bactericidal action or bacteriostatic action) and their potential is mainly related to structure and shape. For example, MCFAs and LCFAs are more active against Gram positive bacteria and unsaturated tend to have more potency, but their efficacy is dependent on external factors. Their action is carried out mainly at pathogen membrane level through disruption of electron transport chain, uncoupling of oxidative phosphorylation, cell lysis, inhibition of enzyme activity, impairment of nutrient uptake, or peroxidation and auto-oxidation [5]. Some are naturally synthetized by the animals in the GIT, and they may be considered as potential energy sources in addition to their activities as acids. Table 3 presents details on the most relevant studies. 

### 4.1. Short-Chain Fatty Acids

The exogenous addition of SCFAs has been undertaken to modulate GIT health status, host immune functions, and general welfare status, and could be used as an energy source for bacteria; in this context, the weakly acid nature and form (coated or uncoated) of the SCFA are also relevant [4]. The presence of SCFAs at certain concentrations is also essential for regulating the presence of pathogenic bacteria (i.e., *Salmonella spp.*, *E. coli*, and *Campylobacter jejuni*). These concentrations depend on various factors, including the intestinal region, diet composition, endogenous microbiota composition, and environmental pH. Some SCFAs (e.g., propionic, acetic, and butyric acids) are naturally present in the intestine as the end products of primary fermentation of non-digestible carbohydrates under anaerobic conditions or secondary fermentation of SCFAs themselves by bacteroides, *Clostridium*, or butyrate-producing bacteria. However, the level and composition of intestinal SCFAs strongly depend on diet and microbiota composition and region intestine [8]. In addition to acid supplementation strategies, some authors have suggested that SCFAs’ concentrations may be beneficially modulated through the diet composition and/or use of probiotics [115,116,117].

#### 4.1.1. Formic Acid

Formic acid (CH_2_O_2_) is a colorless liquid with a pungent odor and a pK_a_ of 3.75 [7]. This has been widely used as a preservative and antibacterial in feed; in pig feed strategy, it is also often used as a growth promoter because of its ability to reduce small intestine, caecum, and colon pH. Partanen et al. investigated the effect of formic acid in growing-finishing pigs and found that it was associated with F/G ratio improvement, faster growth, and a reduced incidence of diarrhea [70]. The effects of formic acid supplementation appear to be period- and dose-dependent; better positive effects were found during the first period post-weaning, whereas long-term supplementation had only a slight effect on microbiota composition at a relatively high dosage [71]. Better improvements were obtained with a formic acid-potassium sorbate blend [71]. Tsiloyiannis et al. showed a positive effect on controlling post-weaning diarrhea [22]. Blank et al. showed that supplementation of formic acid along with microbial phytase could increase the efficacy of the phytase [72]. The application of formic acid with essential oils or other organic acids was reported to increase the apparent fecal digestibility of crude fiber in weaning pigs, and the combination with essential oils also appeared to improve non-starch polysaccharide (NSP) and total carbohydrate digestibility [73]. Other studies found that formic acid did not alter the apparent ileal digestibility of AAs, ileal pH, the VFA concentration, or bacterial populations reported in weanling pigs [74] or the performance of finishing pigs [74,75]. The minimal inhibitory concentration of formic acid is dependent on pH and, at this minimal level, formic acid did not decrease *Salmonella typhimurium* invasion [76].

#### 4.1.2. Acetic Acid

Acetic acid (CH_3_COOH) is the dominant component of intestinal SCFA, with a pKa of 4.76 [8]. Valencia and Chavez tested acetic acid supplementation at different dietary levels of P and concurrent supplementation of phytase, and reported that this protocol significantly improved ADG and improved mineral digestibility (P, Ca) in weaning piglets [77].

#### 4.1.3. Butyric Acid

Butyric acid (C_4_H_8_O_2_; pKa of 4.82) is one of the major energy sources of enterocytes and essential for maintaining the normal metabolism of intestinal mucosa. Butyrate could perform a trophic effect on crypt cell and/or increase the number of microvilli cells and anti-inflammatory effect, regulating cytokine expression. The supplementation into coated form could enhance the acid effect, preventing rapid absorption by epithelia cells [4]. A coated form of butyric acid showed antimicrobial activity by decreasing the fecal shedding of *S. typhimurium* [76]. In contrast, Bosi et al. reported that butyrate supplementation did not interact with the acid form or affect the fecal shedding of *E. coli* [78]. Butyrate supplementation in salt form (sodium n-butyrate) reportedly yielded a higher daily gain and feed efficiency, a lower coliform bacteria count in the ileum, and an increased count of *Lactobacillus spp.* [79]. However, Piva et al. found that a similar supplementation facilitated only the transition from liquid to solid feed in nursery pigs [80]. The supplementation of tributyrin as a butyrate source reportedly also showed beneficial effects, alleviating intestinal injuries and promoting tight-junction formation [81].

#### 4.1.4. Propionic Acid

Propionic acid (C_3_H_6_O_2_) has a pKa of 4.87. Regarding propionic acid dietary supplementation, considering the lowest acidifying potential instead of other OA, the recent bibliography is lacking. Tsiloyiannis et al. showed a positive effect on controlling post-weaning diarrhea and ADFI [22]. Mosenthin et al. reported that this strategy improved the apparent ileal digestibility of several indispensable AAs (arginine, histidine, leucine, phenylalanine, and valine) and decreased the concentration of cecal digesta cadaverine [82].

### 4.2. Medium-Chain Fatty Acids 

MCFAs are saturated 6- to 12-carbon acids, including caproic acid (C6:0), caprylic acid (C8:0), capric acid (C10:0), and lauric acid (C12:0). The undissociated forms are found at pH values between 3 and 6 [118]. MCFA administration seems to provide a positive effect through nutritional, metabolic, antimicrobial, and immune-stimulating effects. MCFAs diffuse into portal blood, where they associate with albumin for direct transport to the liver, where they are used for energy production via mitochondrial ß-oxidation. The contribution of MCFAs as an energy source for the distal GIT epithelium is minor compared with that of butyrate, and only a small portion of the systemic pool of MCFAs is stored in adipose tissues [119]. Undissociated-form MCFAs present in the duodenum can destabilize bacterial membranes and inhibit bacterial lipases [5,120]. MCFAs are typically supplemented into the diet in blends comprising other MCFAs or OA, in order to harness a synergic effect and widen the antibacterial spectrum. MCFAs may also be generated by hydrolysis of medium chain triglycerides (MCTs). MCTs are often used for feed supplementation, but a high dosage may decrease feed palatability and/or create a false sense of satiety due to its rapid oxidation in the liver.

Inconsistent results have been obtained in newborn and lactating piglets, with the results suggesting that MCFA may have toxic effects at high concentrations [121,122]. Regarding supplementation during the weaning phase, Thomas et al. (C6:0 + C8:0 + C10:0) and Mohana Devi et al. (MCFA) reported improvements of ADG, ADFI, and G/F under MCFA supplementation [83,84]. Mohana Devi et al. reported increased glucose levels at weeks 2 and 6 of treatment, along with improved digestion of dry matter and N; these authors obtained similar results with probiotics. Conversely, Cera et al. reported that the feed intake was reduced, but the F/G ratio was improved at 3 weeks after weaning under supplementation with a blend containing MCFAs (C8:0 and C10:0), and that these changes were associated with higher blood triglycerides and lower serum urea concentrations [85]. López-Colom et al. confirmed that MCFA salts from coconut oil (C12:0, C8:0, and C10:0) had antipathogenic effects, such as reducing *Salmonella* in cecum; reducing Enterobacteriaceae and coliform bacteria in the ileum and colon; and increasing the antimicrobial effects of lauric, caprylic, and capric acids beyond the effects seen with long-chain fatty acids (LCFAs) [86]. Marounek et al. reported that MCFA supplementation had a coccidiostatic effect, delaying shedding and shortening the patent periods for cryptosporidial oocysts of *Cryptosporidium parvum* and *Isospora suis* [87]. MCFAs are often supplemented in blends along with other organic acids. According to Zentek et al., the combination of OA (fumaric acid and lactic acid) and MCFA (C8:0; C10:0) could modify the microbiota and prevent post-weaning diarrhea [88]. Han et al. confirmed that MCFAs blends increased growth performance, decreased the diarrhea rate, and enhanced serum immunity [89]. The diarrhea incidence in weanling pigs challenged with *E. coli* was reduced by supplementation with an OA-MCFA (C8:0, C10:0) blend [90]. When the OA-MCFA blend is added with *Enterococcus,* dry matter improvement had been found in finishing pigs [91]. Casellas et al. instead found that minimal supplementation of MCT long chain triglyceride (LCT) could improve the survival of the smallest piglets [92]. There is no evidence that dietary administration of MCTs can affect the reproductive performance of pigs, yield maternal or fetal toxicity, or exhibit teratogenic effects at doses up to 12,500 mg/kg BW/day [123]. MCT supplementation in sows during gestation and lactation reportedly had positive effects on the sows’ body condition score, but did not alter their milk composition; among the offspring, there were improvements in maturity at birth, body glycogen stores, and the survival rate [93,94].

### 4.3. Polyunsaturated Fatty Acids 

The PUFA group comprises sorbic acid, omega-3 (ω-3) and omega-6 (ω-6), and conjugated fatty acids. These are essential fatty acids (EFAs), meaning that they are not endogenously synthesized, but rather must be supplied in the diet. Although the underlying mechanisms are not well understood, an optimal PUFA ratio is essential for the proper maintenance of homeostasis among various biological processes and metabolism [124].

#### 4.3.1. Sorbic Acid

Sorbic acid (C_6_H_8_O_2_) is a polyunsaturated fatty acid (PUFA); it is a colorless solid that acts as a strong acid (pK_a_ 4.76). In the context of feed supplementation, sorbic acid has mainly been used as a preservative in a water-soluble salt form (sodium sorbate, potassium sorbate, calcium sorbate). It is not considered toxic because it is metabolized via ß- and ω-oxidation and its antimicrobial potential is due to the inhibition of enzyme and nutrient transport [18]. Crenshaw et al. reported acidifying potential in high moisture sorghum, but no effect on pig performance [95]. On the other hand, Luo et al. found that, in pigs between 21 and 24 days of age, supplementation of sorbic acid improved ADG, BW, and G/F, while a higher dosage also improved ADG and G/F [96]. The same authors found increases in plasma total serum protein and globulin concentrations, and the expression levels of IGF related genes (those encoding IGF-I, IGF-II, IGF-receptor, and peroxisome proliferator-activated receptor alpha (PPARα)), and linked these changes to an improvement in growth performance [96]. Other authors included sorbic acid in organic acid blends in the diets of entire male pigs, and observed an improvement of intestinal mucosa maturation [97] and the feed conversion ratio, along with lower levels of coliforms, enterococci, and lactic acid producing bacteria (LAB) in the GIT [98].

#### 4.3.2. Omega Fatty Acids

The omega fatty acids, which include members of the ω-3 and ω-6 families, are essential fatty acids. The ω-3 group includes α-linoleic acid (ALA), eicosapentaenoic acid (EPA), and docosahexaenoic acid (DHA), while the ω-6 group includes linoleic acid (LA). Both groups are involved in the inflammatory response and reproductive performance of swine. In swine diets, the ω-6:ω-3 ratio can range from 4:1 to 11:1; this ratio depends on the composition of the diet [4], and proper requirements have not yet been established. Szostak et al. (2016) reported that an optimal ω-6:ω-3 ratio is essential for maintaining the homeostasis of biological processes and metabolism. The same authors reported that an enriched LA and ALA diet reduced the ω-6:ω-3 ratio in the liver to modulate the expression levels of genes involved with energy metabolism, signaling pathways, and the inflammatory response. ω-3 supplementation also influenced the fatty acid content in tissues [99], and decreased the maternal serum level of arachidonic acid [99]. These enhanced tissue levels of fatty acids under ω-3 supplementation were reflected in the fatty acid profiles of sow’s milk when the acid was administered during the last gestation phase, as well as in piglets, which showed increased serum levels of EPA [100]. Supplementation of ω-3 PUFA also affected the endometrial, conceptus, and fetal fatty acid compositions in early pregnancy [101]. The origin of the omega fatty acid has been suggested to affect the results. For example, Eastwood et al. showed that gestating sows fed plant-based diets with ω-6:ω-3 ratios of 5:1 or 1:1 did not affect piglet performance, but improved the conversion of ALA to EPA and enhanced the transfer of ω-3 to piglets via milk, whereas a fish-based diet with a 5:1 ratio was associated with increased pre-weaning mortality, decreased BW, and decreased weaning pig weight [102]. Supplementation with ω-3 from microalgae did not influence the reproductive performance of sows; however, a high dosage reduced the serum triglyceride level during gestation and increased the birth weight of piglets [103]. Estienne et al. did not find that ω-3 supplementation benefited the reproductive performance of gilts; instead, the authors reported that it seemed to accelerate their puberty [125]. DHA supplementation of finishing pigs diet was reported to affect the fatty acid composition of backfat, but not growth performance and carcass traits [104]. Upadhaya et al. found that ω-3 tended to increase ADG and interacted with vit E supplementation and cortisol level [105]. The effect of PUFA supplementation on the immune cells of porcine lung (increasing host resistance and response to respiratory pathogens) was reportedly associated with the influence of PUFA on alveolar macrophages and lymphocytes [106]. DHA supplementation in the form of microalgae did not affect daily gain, feed intake, or feed efficiency; however, at a low dosage, this supplementation increased the ω-3 levels in meat [107].

#### 4.3.3. Conjugated Linoleic Acid

The conjugated linoleic acid (CLA) group includes LA isomers that have antitumoral and immune-enhancing effects. Supplementation of CLA into the diet of sows was reported to affect piglet performance, but not sow reproductive performance. Bontempo et al. and Corino et al. showed that CLA supplementation has a beneficial effect during the partum period (−7 to +7 days) on colostrum Ig (IgG, IgA, IgM), the BW of piglets at weaning, and immune components (IgG) [108,109]. The addition of CLA into the gestation and lactation diet of sows may reportedly be used to enhance the passive immune system of piglets, confer long-term effects during the post-weaning period, and preserve gut health status [110]. Supplementation of CLA during the post-weaning period improved growth performance, lymphocyte proliferation (CD8^+^ cells), and reduced PGE_2_ and interlukin-1β [111]. In weaned piglets challenged with LPS, CLA supplementation showed the capacity to modulate inflammation and inhibit the synthesis of proinflammatory cytokines [112]. The antiviral effect of CLA supplementation was tested by Bassaganya-Riera et al. in pigs challenged with type-2 porcine circovirus (PCV2); the authors found that CLA increased the proliferation of CD8^+^ T cells and suppressed PCV2-specific interferon-γ production in CD4^+^ T cells [113]. The same authors had previously shown that reported that dietary supplementation of CLA for 42 days could prevent disease-associated growth suppression in dirty environmental conditions [114].

## 5. Conclusions

Acids have been widely tested as pig feed additives at different animal ages and diet types and blend. The various groups of acids tested to date have shown clear positive effects on gastric functions’ stimulation, gastric enzyme production, gastric retention time and pH modulation, and gut mucosa integrity and structure growing and nutrient absorptive capacity. Microbiota composition results were affected by acid supplementation through the high antibacterial property. These, together with the modulation potential of the anti-inflammatory response, result in a decrease of enteric disease incidence, enhanced growth performance, and improved feed efficiency. Some acids may also act as energy sources for metabolic processes and meat quality enhancement. A single mode of action could not be considered dominant, but the acid positive effect could be explained by their interaction. This is also because acid blends are often used, further complicating efforts to understand the mode(s) of action; however, such blends may have cascading effects that may enable them to function as non-antibiotic growth promoters and/or reduce the excretion of compounds that burden the environment. To date, however, the obtained results have been inconsistent and appear to be strongly influenced by the treatment dose and time, acid form, diet composition, animal age, and environmental conditions. Certainly, the clearest benefit of acid supplementation is seen during the weaning phase; however, additional studies are needed to fully assess the effect of acid supplementation sows and how a sow’s diet influences her piglets’ performance. Given the improved effects and broadened spectrum of acid blends compared with single acids, further studies should seek to evaluate the impacts of different acids at different phases of life. Within this milieu, it will be important to choose an optimum acid or acid blend/dosage that does not significantly reduce diet palatability. Such optimization of the beneficial effects of acid supplementation may eventually allow pig producers to reduce antibiotic use and prevent the acquisition of tolerance phenomena against acids themselves.

## Figures and Tables

**Table 1:** Summary of studies about the most common inorganic acids used in pig farming.

Compound	Animal Category	Dose	Response	References
Hydrochloric acid	Weaning pigs	0.10%	Improved average daily gain (ADG; 13%), average daily feed intake (ADFI; 12%), and N utilization. Better ADG, ADFI, and gain to feed ratio (G/F) than organic acid (OA) supplementation	[9]
	Weaning pigs	0.06%	Improved N digestibility and retention and growth performance	[12,13]
Calcium chloride	Growing–finishing pigs	4%	Increase of plasma Cl concentration, causing acidosis, and suppressing appetite	[11]
Phosphoric acid			No influence on pig performances	[15]
Inorganic acids (IA) blend	Weaning pigs	2%	Reduction of *E. Coli* shedding	[16]
	Weaning pigs	0.4% OA blend followed by 0.2% IA blend	Improvement of ADG	[17]

ADG: average daily gain; ADFI: average daily feed intake; G/F: gain to feed ratio; IA: inorganic acid; OA: organic acid.

**Table 3:** Summary of studies about the most common fatty acids used in pig farming.

Compound	Animal Category	Dose	Response	Reference
Formic acid	Weaning pigs	1.20%	Positive effect on reduction of post-weaning diarrhea	[22]
	Growing pigs	0.80%	Increased growth performance and feed efficiency; lower diarrhea incidence	[70]
	Weaning pigs	0.14% or 0.64%	Increased average daily gain (ADG), average daily feed intake (ADFI), microbiota diversity, and jejunal expression of C-C motif chemokine ligand 2 (CCL20) at higher dose. Decreased G/F	[71]
	Growing pigs	0.47%	Interaction with phytase supplementation, increased P and Zn digestibility and retention	[72]
	Weaning pigs	0.5% formic acid + essential oils (EO) or 0.5% formic acid + organic acids (OA) (propionic, lactic, citric, sorbic acid)	Increased fecal digestibility of crude fiber, carbohydrates, and non-starch polysaccharides (NSP)	[73]
	Weaning pigs	1.0% wt/wt	No effect on apparent ileal digestibility of diet with low or high buffering capacity	[74]
	Growing pigs	0.80%	Improved growth performance during growing phase, but not during the finishing phase	[75]
	Weaning pigs	10 mM, coated form	Minimal inhibitory concentration (MIC) dependent on pH	[76]
Acetic	Weaning pigs	1.0%	Increased apparent digestibility of P and Ca	[77]
Butyric	Weaning pigs	10 mM, coated form	Reduction of hilA expression, *Salmonella* invasion, and fecal shedding	[76]
	Weaning pigs	0.20% free sodium butyrate or 0.06% protected sodium butyrate	Increased villus height, growth, and surviving rate of *E. coli* challenged pigs	[78]
	Weaning pigs	0.17% sodium n-butyrate	Increased *Lactobacillus* count, ileal microvilli length, and depth of cecal crypts. Reduction of coliform bacteria in ileum, feed and costs	[79]
	Weaning pigs	0.08% sodium n-butyrate	Increased ADG, daily feed intake (DFI), feed efficiency (FE), and live weight (LW)	[80]
	Weaning pigs	0.1% tributyrin	Alleviation of acetic acid-induced intestinal injury. Increased caspase-3 levels, claudin-1 protein, and epidermal growth factor receptor mRNA expression	[81]
Proprionic	Weaning pigs	1.0%	Positive effect on reduction of post-weaning diarrhea	[22]
	Growing pigs	2.0%	Increased apparent ileal digestibility of amino acids (AA; arginine, histidine, leucine, phenylalanine, valine). Reduction of cadaverine concentration in cecal digesta	[82]
Medium-chain fatty acids	Newborn pigs	(1) 0.50% or 1.0% or 2.0% medium chain fatty acids (MCFA) blend 1:1:1 (C6:0 + C8:0 + C10); (2) 1.0% of 4 MCFAs blend (50% C6:0 + 20% lactic acid + 10% or 20% or 30% monolaurin)	(1) Increased ADG, ADFI, and feed efficiency; (2) increased ADFI and ADG with 1.0%	[83]
	Weaning pigs	0.2% MCFA blend	Increased ADG (MCFA), gain to feed ratio (G/F), glucose level in blood, nutrient digestibility of dry matter (DM), N, and energy (MCFA + 0.01% probiotic)	[84]
	Weaning pigs	8% MCFA blend (60% C8:0 + 40% C10:0)	Increased triglyceride concentrations. Reduction of feed intake and superior G/F ratio for 3 weeks, and serum urea concentration	[85]
	Weaning pigs	0.003% (salts form; 48% C12:0 + 18.6% C14:0 + 9.9% C16:0 + 6.8% C18:1 + 6.2% C8:0 + 5.8% C10:0+ 3.6% C18:0 + 1.3% C18:2)	Reduction of *Salmonella* count in cecum, *Enterobacteriaceae* and total coliform in ileum/colon, and intraepithelial lymphocyte counts	[86]
	Weaning pigs	0.05% C8:0 or 0.1% MCFA blend	Increased weight gain. Delaying of cryptosporidial oocysts patent period	[87]
	Weaning pigs	0.2% or 0.4% organic acids (OA) and MCFA (17% fumaric acid +13% citric acid + 10% malic acid + 1.2% MCFA C10:0/C8:0)	Decreased pH digesta, MCFA induced minor changes in microbiota composition instead of OA. Interaction of OA and MCFA to reduce *E. coli* virulence and prevent post-weaning diarrhea	[88]
	Weaning pigs	0.2% OAs blend (C12:0 + butyrates, MCFAs + sorbic acid + phenolic compounds)	Increased *Lactobacillus* and *Faecalibacterium*, growth performance, and serum immunoglobulin (Ig; IgG, IgA). Reduced diarrhea rate	[89]
	Weaning pigs	0.2% or 0.4% OA and MCFA (17% fumaric acid + 13% citric acid + 10% malic acid + 1.2% MCFA C10:0/C8:0)	Increased body weight (BW), ADG, ADFI, and G/F. Reduction of diarrhea incidence	[90]
	Finishing pigs	0.05% or 0.1% OA–MCFA blend (17% fumaric acid + 13% citric acid + 10% malic acid + 12% MCFAs (C8:0/C10:0)	Increased ADG, ADFI, and digestibility of DM	[91]
	Newborn pigs	0.02% MCFA– long chain fatty acids (LCFA) blend (0.9% C6:0 +29% C8:0 + 19% C10:0 + 8% C14:0 + 5% C16:0 + 6% C18:1n -7 + 8% C18:2n – 6 + 1% C18:3n -3)	Higher live weight/body weight ratio; possible toxic effect	[92]
	Sows	Medium chain triglyceride (MCT) (92% C8:0 + 2% C10:0 + 6% C12:0) 9:1 by weight	Improved survival rate to day 21, muscle glycogen, and serum albumin of the smallest pigs	[93]
		10% supplemental fat as MCT source	Increased pig ADG and average pig weaning weight	[94]
Sorbic acid	Weaning pigs	1.0%	Inconsistent results	[95]
		0.05% or 0.2% or 0.4%	Increased ADG, BW, G/F, plasma triglycerides (4.0%), serum protein and globulin, insulin-like growth factor (IGF)-I, IGF-II, IGF-I receptor, and peroxisome proliferator-activated receptor α (PPARα) gene mRNA expression	[96]
		0.5% OA-EO blend (0.08% sorbic acid + 0.13% citric acid + EO)	Increased intestinal mucosa maturation and modulation of local and systemic inflammatory. Reduced ileum short circuit current (I_sc_) in ileum, ileal gene expression of interleukin-12 (IL-12), transforming growth factor- β (TGF-β), and IL-6	[97]
	Growing pigs	OAs blend (1% formic acid + 0.85% benzoic acid + 0.85% sorbic acid + 1.2% coated Ca-butyrate)	Increased feed conversion ratio (FCR). Reduced coliforms, enterococci, and lactic acid counts	[98]
Omega fatty acids	Weaning pigs	5.0% fish oil	Increased ω-3 in tissues (muscle, adipose tissue, heart, liver, kidney). Reduction of arachidonic acid (heart, liver, kidney), lauric and myristic acids (muscle, adipose tissue)	[99]
	Sows	3.5% or 7.0%	Increased ω-3 polyunsaturated fatty acids (PUFA) in sow serum and milk. Decreased sow serum and milk arachidonic acid. Newborn piglets enriched in ω-3 PUFA, EPA serum level	[100]
	Sows	1.5% protected fatty acids (FA)	Increased DHA in fetus, chorioallantois, endometrium, blood EPA and DHA, extraembryonic EPA, and embryonic tissues DHA	[101]
	Sows	ω-6/ω-3 of 9:1/5:1/1:1 in plant oil-based diets; 5:1 fish oil-based diet (5% crude fat diet)	Increased weaning weight and ADG (from birth to weaning) with 9:1 and 5:1 plant based diet, sow serum ω-3 (1:1 plant-based, 5:1 fish-based), EPA in pre-suckle pigs serum (1:1 plant-based, 5:1 fish-based), in post-suckle pigs serum ALA (1:1 plant-based) and EPA and DHA (5:1 fish-based). Reduced sow feed consumption during lactation, pig birth weight, pig weaning weight (5:1 fish-based)	[102]
	Sows	3.5 or 7.0 or 14.0 or 28.0 g/day (seaweed meal 12% DHA)	Increased pigs’ birth weight (28.0 g/d). Reduced serum level of triglycerides (28.0 g/d)	[103]
	Finishing pigs	1.0% unextracted *Aurantiochytrium limacinum* (18% DHA)	Increased EPA, DHA, ω-3, ω-3:ω-6 in *Longissimus Lumborum* and DHA and ω-3 in backfat	[104]
	Growing pigs	0.75% protected ω-3	Tendency to increase ADG. Interaction between ω-3 and Vit E on cortisol levels	[105]
	Growing pigs	10.5% corn oil (CO; enriched in linoleic acid (18:2, n-6)), linseed oil (LO; enriched in α-linolenic acid (18:3, n-3)), menhaden oil (MO; enriched in eicosapentaenoic (20:5; n-3) and docosahexaenoic (22:6; n-3) acids), linseed + corn oil (1:1; LC), and menhaden + corn oil (1:1; MC)	Increased levels of alveolar macrophage (mφ) tumor necrosis factor-α (TNF-α) production were higher for LC- and MC-fed pigs, lipopolysaccharide (LPS)-stimulated LC and MC mφs produced more TNF than mφs macrophages from pigs receiving the CO, LO and MO. CO and LC diets had higher levels of leucine aminopeptidase; LPS-stimulated mφs from the CO, MO, and LC dietary groups produced more nitrite than mφs from MC-fed pigs. Alveolar lymphocytes from pigs receiving the MC diet produced more T cell growth factors than LO and MO mφs. Alveolar mφs from the different dietary groups did not differ in their capacity for non-immune-mediated phagocytosis of fluorescent latex beads	[106]
	Weaning pigs	0.125 or 0.250 kg/pig docosahexaenoic acid (DHA)	Increased eicosapentaenoic acid (EPA), docosahexaenoate, and docosapentaenoate ω-3 and ω-6 in ham, loin and shoulder (0.250 g/pig), docosapentaenoate ω-6 and docosahexaenoate (0.125 g/pig)	[107]
Coniugated linoleic acid (CLA)	Sows and piglets	0.5%	Increased colostrum eicosenoic and eicosatrienoic acids and IgG, sow serum leptin, IgG and lysozyme, and in nursing piglets from CLA-fed sows serum lysozyme and IgG, and piglets IgG titer. Reduction of palmitoleic and γ-linolenic acid were significant for colostrum, piglet serum α-1 acylglicoprotein	[108]
	Sows	0.5%	Increased piglet BW at weaning, serum thyroxine concentration in sows, colostrum IgG, IgA, and IgM titers	[109]
	Sows and piglets	2.0%	Increased piglets’ ADFI (17–28 d), serum IgG, and IgA from treated sows. Reduced development of scours in *E. coli* challenged piglets, and piglets’ intestinal mucosa inflammation	[110]
	Weaning pigs	1.0% or 2.0% or 3.0%	Increased ADG, ADFI, G/F, lymphocyte proliferation, ovalbumin antibody production, and CD8^+^ lymphocyte population. Reduction of prostaglandin E2 and IL-1β	[111]
	Weaning pigs	2%	Alleviated growth depression and prevention of the elevations in production and mRNA expression of proinflammatory cytokines (IL-6) and TNF-α induced by the LPS challenge. Increased expression of IL-10 and PPARγ in spleen and thymus	[112]
	Weaning pigs	1.33%	Interaction with porcine circovirus (PCV) 2 to increase the proliferation of CD8+ T cells and to suppress PCV2-specific interferon (IFN)-γ production in CD4+ T cells	[113]
	Weaning pigs	0.67% or 1.33% or 2.0%	Decreased ADG and ADFI with increase in CLA; after adaptation ADG and G/F increased, percentages of CD8+ lymphocytes (after 42d) and ADFI decreased	[114]

AA: amino acid; ADFI: average daily feed intake; ADG: average daily gain; BW: body weight; CLA: conjugated linoleic acid; DFI: daily feed intake; DHA: docosahexaenoic acid; DM: dry matter; EPA: eicosapentaenoic acid; EO: essential oils; FA: fatty acid; FCR: feed conversion ratio; FE: feed efficiency; G/F: gain to feed ratio; Ig: immunoglobulin; I_SC_: ileum short circuit current; IGF: insulin-like growth factor; IFN: interferon; IL: interleukin; LCFA: long chain fatty acids; LPS: lipopolysaccharide; LW: live weight; MCFA: medium chain fatty acid; MCT: medium chain triglycerides; MIC: minimal inhibitory concentration; NSP: non starch polysaccharides; OA: organic acid; PCV: porcine circovirus; PPAR: peroxisome proliferator-activated receptor; PUFA: poly-unsaturated fatty acid; TGF: transforming growth factor; TNF: tumor necrosis factor.
